# A Clinical, Neuropathological and Genetic Study of Homozygous A467T *POLG*-Related Mitochondrial Disease

**DOI:** 10.1371/journal.pone.0145500

**Published:** 2016-01-06

**Authors:** Sanjeev Rajakulendran, Robert D. S. Pitceathly, Jan-Willem Taanman, Harry Costello, Mary G. Sweeney, Cathy E. Woodward, Zane Jaunmuktane, Janice L. Holton, Thomas S. Jacques, Brian N. Harding, Carl Fratter, Michael G. Hanna, Shamima Rahman

**Affiliations:** 1 UCL Institute of Neurology and National Hospital for Neurology and Neurosurgery and the MRC Centre for Neuromuscular Diseases, Queen Square, London WC1N 3BG, United Kingdom; 2 UCL Institute of Neurology and National Hospital for Neurology and Neurosurgery, Queen Square, London WC1N 3BG, United Kingdom and Department of Basic and Clinical Neuroscience, Institute of Psychiatry, Psychology and Neuroscience, King’s College London SE5 8AF, United Kingdom; 3 Department of Clinical Neurosciences, UCL Institute of Neurology, London NW3 2PF, United Kingdom; 4 Mitochondrial Research Group, Genetics and Genomic Medicine, UCL Institute of Child Health, London WC1N 1EH, United Kingdom; 5 Department of Neurogenetics, UCL Institute of Neurology and National Hospital for Neurology, Queen Square, London WC1N 3BG, United Kingdom; 6 Division of Neuropathology, UCL Institute of Neurology and National Hospital for Neurology, Queen Square, London WC1N 3BG, United Kingdom; 7 Developmental Biology and Cancer Programme, UCL Institute of Child Health and Department of Histopathology, Great Ormond Street Hospital for Children Foundation Trust, London WC1N 1EH, United Kingdom; 8 Division of Neuropathology, The Children’s Hospital of Philadelphia, Philadelphia, Pennsylvania, United States of America; 9 Oxford Medical Genetics Laboratories, Oxford University Hospitals NHS Trust, Churchill Hospital, Oxford OX3 7LE, United Kingdom; 10 Metabolic Unit, Great Ormond Street Hospital, London WC1N 3JH, United Kingdom; Medical University of South Carolina, UNITED STATES

## Abstract

Mutations in the nuclear gene *POLG* (encoding the catalytic subunit of DNA polymerase gamma) are an important cause of mitochondrial disease. The most common *POLG* mutation, A467T, appears to exhibit considerable phenotypic heterogeneity. The mechanism by which this single genetic defect results in such clinical diversity remains unclear. In this study we evaluate the clinical, neuropathological and mitochondrial genetic features of four unrelated patients with homozygous A467T mutations. One patient presented with the severe and lethal Alpers-Huttenlocher syndrome, which was confirmed on neuropathology, and was found to have a depletion of mitochondrial DNA (mtDNA). Of the remaining three patients, one presented with mitochondrial encephalomyopathy, lactic acidosis and stroke-like episodes (MELAS), one with a phenotype in the Myoclonic Epilepsy, Myopathy and Sensory Ataxia (MEMSA) spectrum and one with Sensory Ataxic Neuropathy, Dysarthria and Ophthalmoplegia (SANDO). All three had secondary accumulation of multiple mtDNA deletions. Complete sequence analysis of muscle mtDNA using the MitoChip resequencing chip in all four cases demonstrated significant variation in mtDNA, including a pathogenic *MT-ND5* mutation in one patient. These data highlight the variable and overlapping clinical and neuropathological phenotypes and downstream molecular defects caused by the A467T mutation, which may result from factors such as the mtDNA genetic background, nuclear genetic modifiers and environmental stressors.

## Introduction

Mutations in the nuclear gene *POLG*, which encodes the catalytic subunit of DNA polymerase γ (Pol γ), the sole enzyme responsible for DNA replication and repair in mitochondria, result in a highly heterogeneous group of mitochondrial disorders [1]. The phenotypic spectrum of *POLG*-related mitochondrial disease includes progressive external ophthalmoplegia (PEO), sensory and cerebellar ataxia, encephalopathy, neuropathy, focal and generalised epilepsy, dysarthria, distal myopathy, Parkinsonism, liver disease and premature ovarian failure [2–10]. Although several distinct phenotypes have been reported in association with *POLG* mutations including childhood-onset Alpers-Huttenlocher syndrome, autosomal recessive and dominant of forms of PEO, myoclonic epilepsy, myopathy and sensory ataxia (MEMSA) and the ataxia-neuropathy spectrum (ANS) disorders, current thinking suggests that these previously defined syndromes are not discrete clinical entities but rather overlap considerably and lie on a continuum. One of the major challenges ahead is to delineate the full spectrum of *POLG*-related disease.

The c.1399G>A mutation in exon 7 of *POLG* produces an alanine to threonine substitution (A467T) at a highly conserved site and is the most frequent pathogenic mutation in *POLG*-related mitochondrial disease. Exon 7 encodes the spacer domain of the polymerase, the function of which is largely unknown; although mutagenesis of this conserved region in the fruit fly protein has been shown to alter the activity, processivity and DNA-binding affinity of the enzyme [11]. A467T exhibits common European ancestry with a carrier frequency of approx. 0.2% to 0.3% in mixed populations of European origin (http://exac.broadinstitute.org; http://evs.gs.washington.edu/EVS) although the carrier frequency has been reported to be as high as 1.3% to 1.4% in Belgian and British populations respectively [3, 12]. Assuming Hardy-Weinberg equilibrium, the predicted homozygote rate would be 1 in 500,000 to 1 in 1,000,000 for a carrier frequency of 0.2% to 0.3%. The A467T mutation is functionally recessive and is usually found *in trans* with another *POLG* mutation, although homozygous A467T mutations do occur (Human DNA Polymerase Gamma Mutation Database, http://tools.niehs.nih.gov/polg/). *In vitro* studies have revealed that the A467T mutant enzyme exhibits a profound reduction in polymerase activity and processivity as a result of impaired interaction with the accessory subunit of the enzyme encoded by *POLG2* [11, 13].

The reasons underlying the variability in clinical spectrum and phenotypic severity associated with the homozygous A467T substitution remain unclear. We studied four unrelated patients with homozygous A467T *POLG* mutations and assessed them longitudinally to characterise the clinical spectrum of A467T-related disease. We undertook molecular genetic studies including MitoChip resequencing analysis, to consider the role of genetic modifiers on phenotype, and performed detailed pathological analysis of muscle, brain and liver samples. We present the results of our investigations and consider the mechanisms by which homozygous A467T mutations give rise to such diverse phenotypes.

## Patients and Methods

### Case histories

We investigated four patients of European descent with homozygous A467T *POLG*-related mitochondrial disease. Clinical details are summarised in Table 1 and in the clinical vignettes presented below.

**Table 1:** Summaries of the case histories of the four patients.

	Patient 1	Patient 2	Patient 3	Patient 4
**Age at presentation (years)**	3	6	20	24
**Age at death (years)**	5.5	Alive at 16	44	Alive at 31
**Symptoms at presentation**	Seizures	Encephalitis-type presentation	Diplopia	Seizures
**Clinical phenotype**	Alpers-Huttenlocher	MEMSA+	SANDO	“MELAS-like”
**Blood/CSF results**	GGT 170 IU/l (reference <20 IU/L), AST 490 IU/L (reference range 5 to 45 IU/l)	↑lactate 2.6mmol/L (<2) Liver function normal; ↑Plasma alanine 537 mcmol/L (150–450); ↓Plasma arginine 28 mcmol/L (40–120); CSF lactate 1.6mmol/L (<2); ↑CSF protein 1.32 g/L (0.15–0.6); CSF 5MTHF 29 (46–120)	↑ lactate 2.3mmol/L (< 1.65); CK 329	Normal lactate; Normal CSF exam
**Neurophysiology**	-	EEG: Intermittent runs of rhythmic delta activity; CS: sensory neuropathy affecting legs	NCS: Severe axonal neuropathy	NCS: Moderately severe axonal sensory motor neuropathy
**Radiology**	Chronic grey matter ischaemia	MRI: Bilateral occipital lesions around calcarine sulci	-	MRI: Right occipital infarct
**Neuropathology**	Cortical degeneration in the occipital and parietal lobes, typical of PNDC. Bilateral hippocampal sclerosis. Hepatic microsteatosis	Brain biopsy: Non-specific; Muscle histology: COX-negative fibres	Muscle histology: ↑ no. of ragged red fibres and > 10 COX-negative fibres	Muscle histology: Ragged red fibres and COX-negative fibres and marked variation in fibre size with scattered groups of atrophic fibres.
**Muscle Respiratory Chain enzymes**	-	Complex I 0.126 (0.104–0.268); Complex II 0.159 (0.040–0.204); Complex IV 0.026 (0.014–0.034)	Complex I 0.170 (0.104–0.268); Complex II 0.077 (0.040–0.204); Complex IV 0.024 (0.014–0.034)	-

**Key:** 5MTHF, 5-methyltetrahydrofolate; AST, aspartate aminotransferase; COX, cytochrome oxidase; EEG, electroencephalogram; GGT, gamma-glutamyltranspeptidase; MELAS, mitochondrial encephalomyopathy, lactic acidosis, stroke-like episodes; MEMSA, myoclonic epilepsy, myopathy, sensory ataxia; NCS, nerve conduction studies; PNDC, progressive neuronal degeneration of childhood; SANDO, Sensory Ataxia Neuropathy Dysarthria Ophthalmoplegia.

#### Patient 1: Alpers-Huttenlocher syndrome

Patient 1 was the second child of healthy unrelated parents and was born at full term. Speech and motor delay were noted at the age of two years. At three years she developed epilepsy and six months later experienced migrainous attacks associated with vomiting, vertigo and transient left-sided weakness. Both electroencephalogram (EEG) and electromyogram (EMG) were normal. At the age of four years she suffered a 10-day episode of headache and vomiting culminating in seizures and coma. Computed tomography (CT) of the brain was normal and EEG showed post-ictal changes only. At the age of five years she developed status epilepticus. An EEG revealed bilateral post-ictal activity with slow wave activity in the left hemisphere. A CT brain demonstrated mild cerebral oedema and magnetic resonance imaging (MRI) brain appearances suggested chronic ischaemia of the grey matter. A month later she was noted to have nystagmus, hypotonia of the lower limbs and absent knee jerks. Liver function derangement was noted (Table 1). A clinical diagnosis of Alpers-Huttenlocher syndrome was made. Her seizure disorder continued to worsen and she died at the age of five years and six months. Post-mortem brain and liver histology subsequently confirmed the diagnosis of Alpers-Huttenlocher syndrome (see results below).

#### Patient 2: “MEMSA +”

Patient 2 was born at full term and had normal development until the age of 6 years when she presented with an encephalopathic illness consisting of impaired consciousness, vomiting and generalised tonic-clonic seizures. One week later she developed a left homonymous hemianopia. MRI brain demonstrated an enhancing lesion in the right occipital lobe and an EEG demonstrated frequent occipital lobe discharges. A repeat MRI showed persistence of the right-sided lesion, with additional left sided occipital lobe swelling. A brain biopsy performed 9 weeks into admission revealed non-specific findings, with no evidence of inflammation, vasculitis, malformation or metabolic disorder. She remained stable until the age of 13 years when she developed stimulus sensitive myoclonus, tremor and a progressive cerebellar ataxia. She has remained cognitively intact throughout. Further investigations at the age of 15 years revealed mildly elevated blood lactate and alanine levels and a sensory axonal peripheral neuropathy (Table 1). An MRI brain revealed bilateral occipital lobe infarcts. Muscle biopsy demonstrated cytochrome-*c* oxidase (COX) negative fibres (see results below) and lipid deposition. Spectrophotometric analysis of respiratory chain enzymes showed low-normal complex I activity. Examination aged 17 years revealed gaze-evoked nystagmus. Her vision was reduced to finger counting on the left and hand movements on the right. Fundoscopy demonstrated bilateral optic atrophy. In addition, she exhibited a hand tremor, stimulus-sensitive myoclonus, head titubation, and gait ataxia. Motor strength was normal, reflexes were present and symmetrical and plantar responses were flexor.

#### Patient 3: SANDO

Patient 3 presented at the age of 20 years with diplopia and bilateral ptosis. Over the next five years he developed dysphagia, slurred speech and an unsteady gait. In his twenties he experienced a tingling sensation in his hands and feet, which in his thirties progressed to involve his legs, trunk and arms. His mother and maternal uncle were also noted to have ‘droopy eyelids’. Neurological examination at the age of 44 years demonstrated bilateral ptosis and limitation of eye movements in all directions of gaze. His speech was dysarthric. Tone and power were normal in all muscle groups. Sensory testing revealed a reduction in pinprick, light touch and temperature sensation in his hands and below the mid-shin level bilaterally. Romberg’s test was positive. His gait was broad-based and ataxic. Nerve conduction studies revealed an axonal, primarily sensory peripheral neuropathy. Muscle histology demonstrated several ragged red fibres and more than 10 COX-negative fibres (see results below). His clinical presentation was consistent with a diagnosis of SANDO.

#### Patient 4: “MELAS-like”

Patient 4 presented at the age of 24 years with recurrent generalised seizures. At the time, she complained of an occipital headache and was noted to be drowsy and confused. She subsequently developed left-sided weakness with sensory loss, and examination also revealed a left homonymous hemianopia suggestive of a stroke-like episode. An MRI brain scan demonstrated a right occipital infarct. A few months later, she was noted to have increased jerking movements of her left arm suggestive of epilepsia partialis continua with dystonia, which was refractory to treatment. Transpial resection brain surgery was unsuccessful. She developed an asymptomatic axonal neuropathy, deafness and myopathic weakness. The salient features on examination were: bilateral ptosis, ophthalmoparesis, a dense left homonymous hemianopia, dysarthric speech, increased tone with clawing of the left hand, and distal muscle weakness. In addition, Romberg’s test was positive and she walked with a wide-based gait. Nerve conduction studies confirmed the presence of a moderately severe axonal sensory motor neuropathy and a muscle biopsy demonstrated the typical findings of a mitochondrial myopathy with ragged red fibres and COX-negative fibres (see results below).

### Neuropathology

Samples of formalin-fixed paraffin embedded (brain, liver) and fresh frozen (muscle, liver) tissues were examined by standard diagnostic protocols.

### Biochemical analysis

Spectrophotometric assays of respiratory chain enzyme complexes were performed in homogenised snap-frozen skeletal muscle biopsy specimens using standard methods [14].

### Molecular genetics

Total genomic DNA was extracted from blood and muscle biopsy specimens from all four patients using standard protocols. Testing for the A467T mutation was achieved by amplification of exon seven of the *POLG* gene using flanking exonic primers followed by automated Sanger sequencing of DNA extracted from peripheral blood leukocytes. MtDNA was assessed for large-scale rearrangements and depletion using long range PCR and Southern blot analysis of total genomic DNA from muscle. The entire mtDNA sequence was analysed in muscle using the GeneChip^®^ Human Mitochondrial Resequencing Array 2.0 (Affymetrix) according to previously published methods [15]. The ~16.6 kb mitochondrial genome was amplified in two fragments using the Expand Template Long PCR Kit from Roche Diagnostics (Mannheim, Germany) according to the manufacturer's protocol. PCR primers and cycling conditions are available on request. Concentration of DNA in the long PCR products was determined using nanodrop spectrophotometry, and equimolar concentrations of the two PCR products were pooled. These were digested with DNAseI. Prehybridisation, hybridisation, washing and scanning of the GeneChip^®^ were performed according to the Affymetrix CustomSeq Resequencing protocol. Sequences were analysed using GSEQ 4.2 software. SNPs were automatically called by GSEQ and presented in a SNP viewer format. Haplogroups were assigned using MitoTool software (http://www.mitotool.org/) by examination of key defining polymorphisms.

The study was approved and performed under the ethical guidelines issued by the Joint National Hospital for Neurology and Neurosurgery and University College London—Institute of Neurology Ethics Committee for clinical studies, with written informed consent obtained for all subjects for genetic studies, diagnostic tests and medical procedures. In the case of minors, informed written consent was obtained from their guardian on their behalf. All persons providing consent were clinically assessed to have capacity.

## Results

### Pathology

#### Patient 1

**Fig 1:**
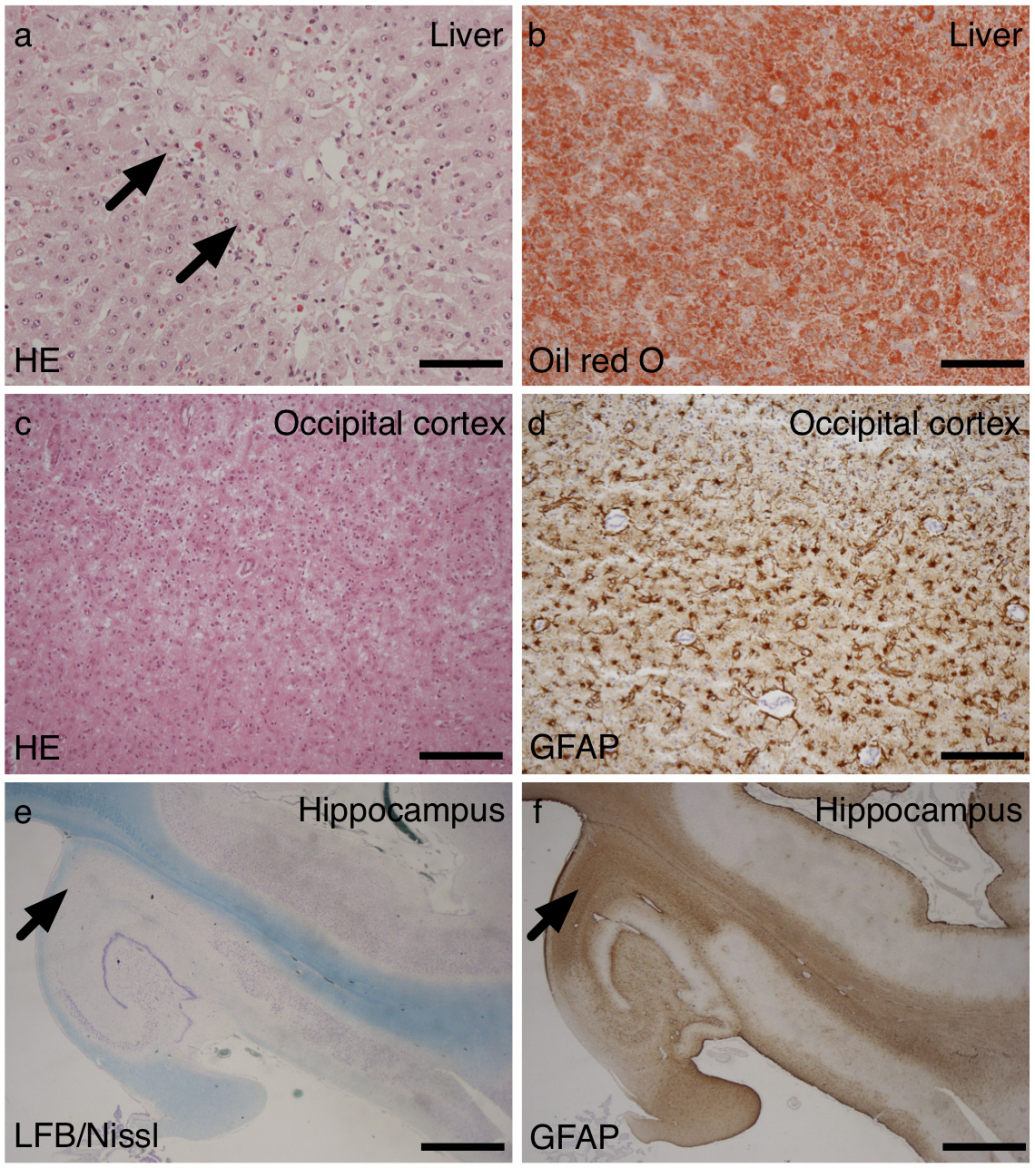
Liver and brain pathology for Patient 1. Post-mortem liver samples from patient 1 (a and b) showed perivenular foci of enlarged hepatocytes with fine vacuolation (arrows). On lipid staining with oil red O (b) of frozen sections there was diffuse lipid deposition. Sample of the cerebral cortex from the occipital lobe showed full thickness neuronal loss with vacuolation and astrocytosis (c and d). Samples of the hippocampi (e and f) showed segmental neuronal loss, most marked from CA1 (arrow) and gliosis in a similar pattern (f-GFAP). Scale bars, a and b = 100 μm; c and d = 200 μm; e and f = 2 mm.

The liver showed patchy, predominantly perivenular areas of enlarged finely vacuolated hepatocytes and some congestion. On Oil red O staining, there was marked diffuse increase in lipid deposition in a microsteatotic pattern (Fig 1a and 1b).

The fixed weight of the post-mortem brain was 1130 g. The external examination was unremarkable. On coronal slicing, there were no significant abnormalities. In particular there was no macroscopic evidence of focal cortical lesions. On histology (Fig 1c–1f), there were focal areas of cortical damage, most prominent in the occipital lobes but also extending in to the parietal lobes. The most severe foci (the occipital lobe) were characterized by neuronal loss, vacuolation of the neuropil (imparting a reticulated appearance) and astrocytosis (confirmed on GFAP (glial fibrillary acidic protein) staining). The more mildly affected areas (the parietal lobes) showed superficial astrocytosis, affecting layers I and II of the cerebral cortex. The white matter was well preserved with only a few foci of pallor on luxol fast blue (LFB) staining. The thalamus showed some patchy vacuolation, neuronal loss, acute neuronal ischaemia and gliosis. The nuclei of the basal ganglia were unremarkable. Both hippocampi showed segmental neuronal loss of neurons in CA1 with less severe loss in CA3 and CA4. CA2 and the dentate gyrus were well preserved. Unequivocal granule cell dispersion was not seen. GFAP immunohistochemistry showed dense gliosis particularly in CA1 and to a lesser extent CA4. The cerebellum was well preserved with a few short gaps in the Purkinje cell layer. There was a prominent Bergman gliosis on GFAP staining. The brainstem was unremarkable.

#### Patient 2

**Fig 2:**
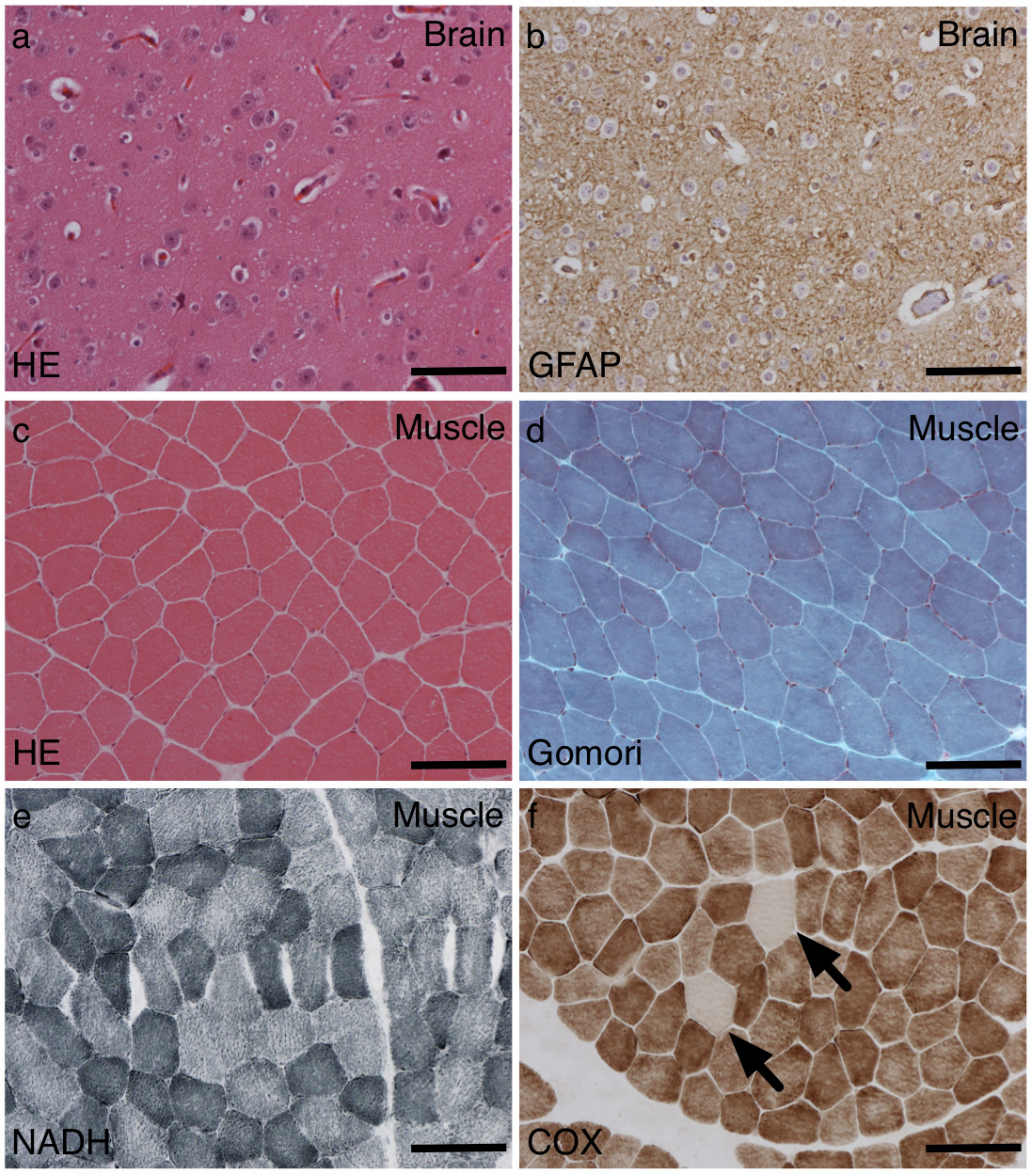
Brain and muscle pathology for patient 2. A brain biopsy from patient 2 showed a little neuropil vacuolation (a) and cortical gliosis (b) but no specific diagnostic features. A muscle biopsy showed scattered cytochrome oxidase (COX)-negative fibres (f—arrows) but no other myopathic features (c) and no ragged red (d) or blue (e) fibres. Scale bars = 100 μm.

A brain biopsy taken at age 6 year showed cerebral cortex and a small amount of superficial white matter. The cortex was well populated with neurons, which showed no specific pathological abnormalities. There was some fine vacuolation of the neuropil but there were no diagnostic features. There was a diffuse cortical astrocytosis revealed by GFAP staining (Fig 2a and 2b).

A muscle biopsy taken at age 15 years showed a normal variation in fibre size with no excess internal nuclei and no destructive features (no necrosis or regeneration). There was no evidence of significant endomysial fibrosis. There were no ragged red fibres. There was a little prominent lipid deposition on Oil red O. Nicotinamide adenine dinucleotide tetrazolium reductase (NADH-TR) histochemistry showed preserved myofibre architecture. There were no ragged blue fibres on succinate dehydrogenase (SDH) histochemistry. There were scattered COX-negative fibres. Fibre typing was within normal limits for the site (Fig 2c–2f).

#### Patient 3

**Fig 3:**
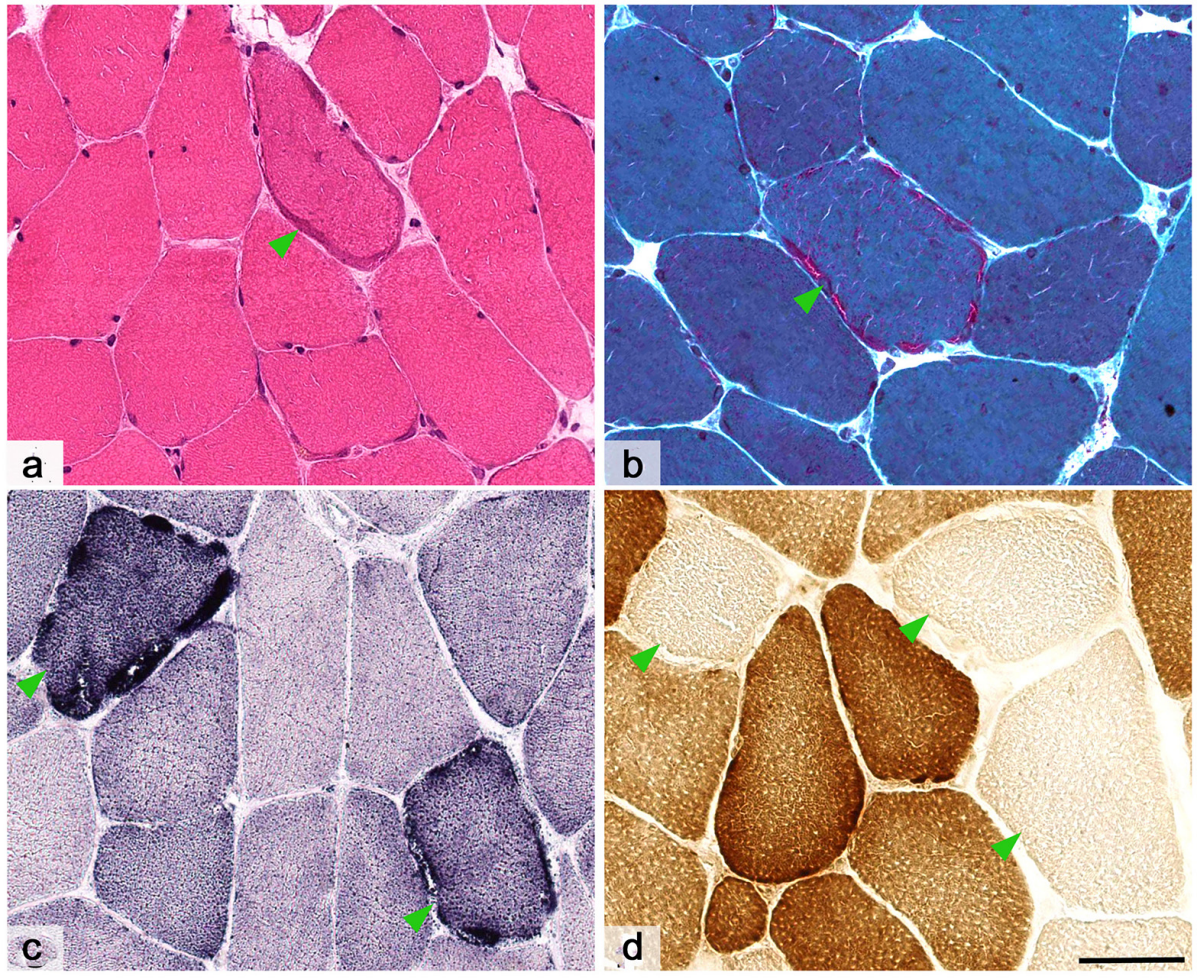
Muscle pathology for patient 3. Haematoxylin-Eosin stained section (A) showed mild variation in fibre size and several fibres with peripheral accumulation of mitochondria (arrowhead). Gomori trichrome preparation (B) accentuated ragged red fibres (arrowhead) and that for Succinic dehydrogenase (C) showed many ragged blue fibres (arrowheads). COX histochemical preparation (D) revealed frequent COX-deficient fibres (arrowheads) in keeping with mitochondrial myopathy. Scale bar = 50 μm.

A deltoid muscle biopsy taken at the age of 43 years revealed mild variation in fibre size with occasional angular atrophic fibres and several fibres with internal nuclei but no increase in nuclear bag fibres or endomysial connective tissue. There was no evidence of regeneration, necrosis or inflammation. Glycogen and lipid content was normal and fibre typing showed a normal fibre distribution. There were frequent ragged red fibres and ragged red fibre equivalents on haematoxylin and eosin (H&E), Gomori trichrome and SDH histochemical preparations. More than 10 COX-deficient fibres were identified in the biopsy (Fig 3a–3d).

#### Patient 4

**Fig 4:**
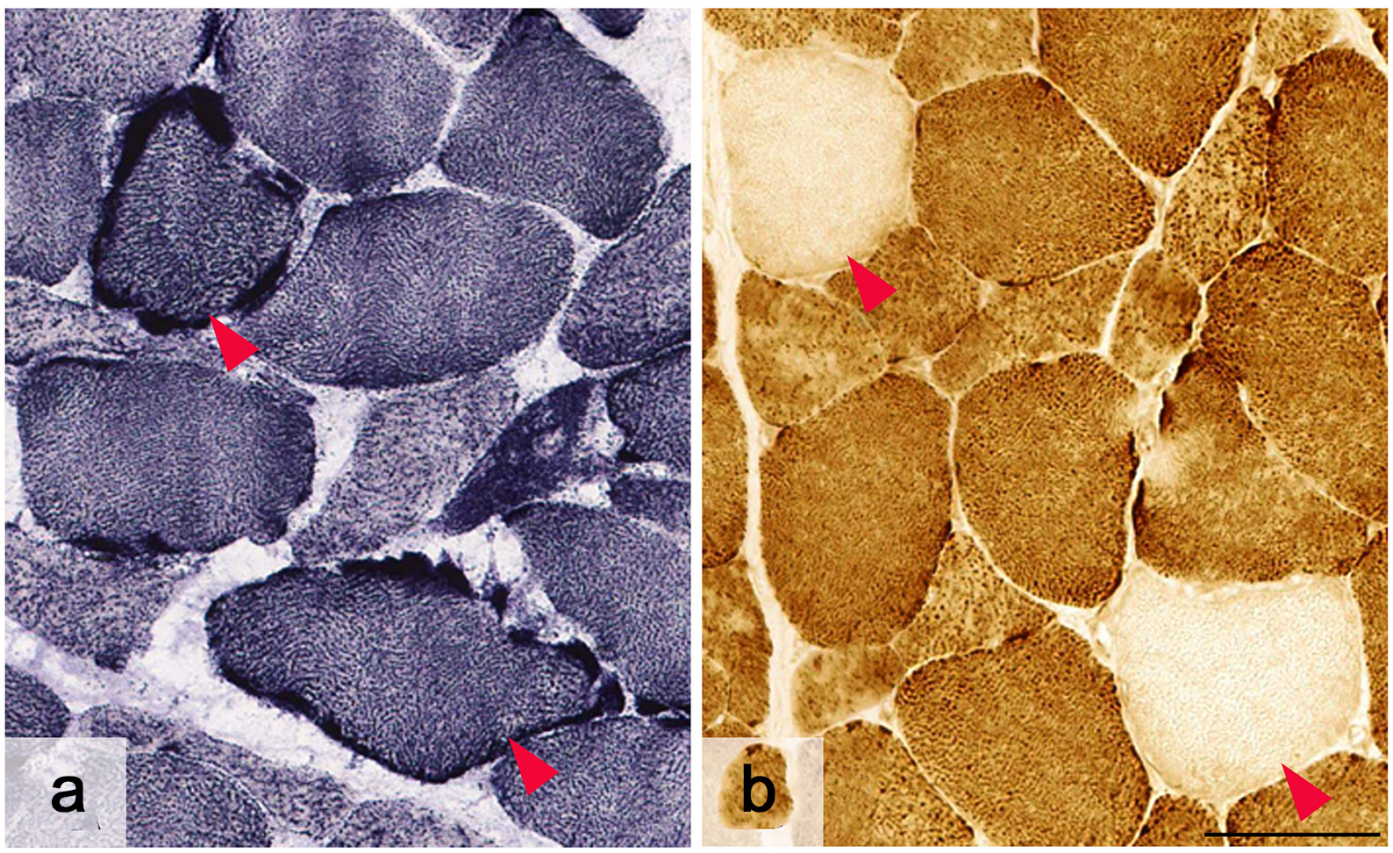
Muscle pathology for patient 4. Succinic dehydrogenase revealed frequent ragged blue fibres (A—red arrowheads) and there were numerous COX-deficient fibres (B—red arrowheads) in keeping with mitochondrial myopathy. Scale bar = 50 μm.

A deltoid muscle biopsy taken at the age of 30 years revealed mild variation in fibre size with no significant endomysial or perimysial fibrosis, no regeneration or degeneration, no vacuoles and no inflammation. Although ragged red fibres were scarce on Gomori trichrome, frequent ragged blue fibres were seen on SDH histochemistry and COX-deficient fibres were numerous (Fig 4a and 4b).

### Biochemistry

Results of respiratory chain enzyme measurements in skeletal muscle are presented in Table 1.

### Molecular genetics

**Fig 5:**
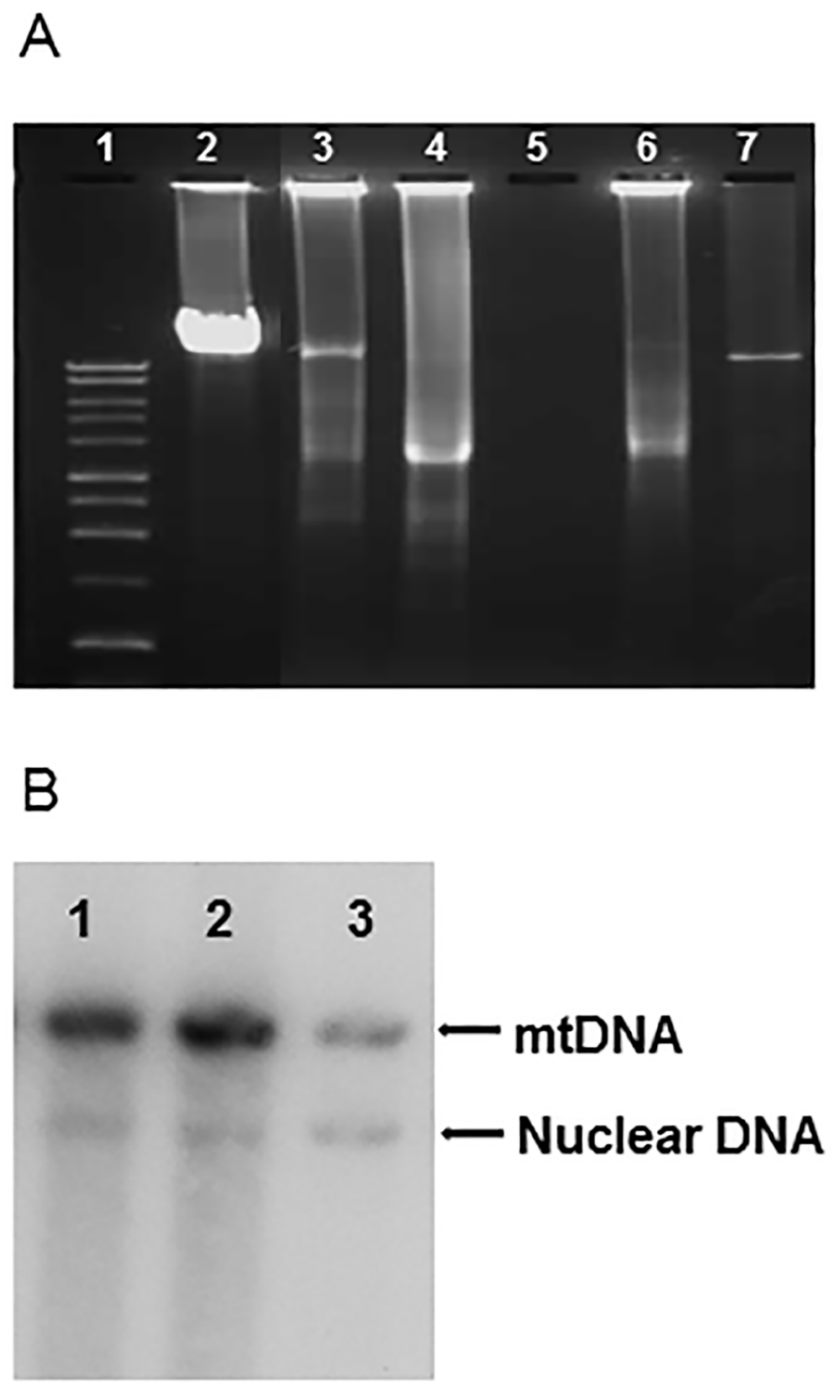
Long range PCR and Southern Blot analysis. **A.** Long PCR of muscle mt DNA from the four patients. **1,** 1kb ladder; **2,** Muscle negative control; **3,** Patient 2; **4,** Patient 3; **5,** water control; **6,** Patient 4; **7,** patient 1. **B.** Depletion of mtDNA obtained from liver of patient 1. **1,** control; **2,** control; **3,** patient 1.

Long-range PCR of mtDNA from patients 2, 3 and 4 revealed multiple deletions of mtDNA (Fig 5a), whilst only a full-sized wild-type mtDNA molecule was detected in patient 1. Southern blot analysis demonstrated that patient 1 had depletion of mtDNA in liver (25% residual mtDNA; Fig 5b), muscle (33% residual mtDNA; not shown) and heart (55% residual mtDNA; not shown) but normal levels of mtDNA in the kidney (not shown). Sequencing of the *POLG* gene identified a homozygous G to A change at nucleotide position (np) 1399 in exon 7 (c.1399G>A; p.A467T) in all four individuals.

Mitochip re-sequencing analysis of the entire mtDNA molecule revealed a large number of variants in each of the four patients (identified variants are summarised in Table 2) with considerable variation between individuals. Patient 4 had an A to G point mutation at np 13528 in the complex 1 *MTND5* gene which has been previously reported to be pathogenic in this patient [16]. In addition, Patient 4 harboured an A>G change at np 12307, which has never previously been reported and a C>T change at np 13565. The first is in the *MTTL2* gene encoding the transfer RNA for leucine (CUN), whilst the second results in a serine to phenylalanine substitution in the ND5 subunit of complex I. Patient 1 harboured a G>A transition at np 14279 which results in a serine to leucine substitution in the ND6 subunit of complex I. This has previously only been reported in 1/2704 individuals (http://www.mtdb.igp.uu.se/). Haplotype data for each patient is presented in Table 2.

**Table 2:** Muscle mtDNA resequencing data of the four patients.

MtDNA base position	Reference sequence	Patient 1	Patient 2	Patient 3	Patient 4	Amino Acid change	Locus
73	A	A	A	**G**	**G**	Non-coding	MT-D Loop
152	T	**C**	T	**C**	**C**	Non-coding	MT-D Loop
195	T	T	T	**C**	**C**	Non-coding	MT-D Loop
263	A	**G**	**G**	**G**	**G**	Non-coding	MT-D Loop
456	C	C	**T**	C	C	Non-coding	MT-D Loop
477	T	**C**	T	T	T	Non-coding	MT-D Loop
499	G	G	G	G	**A**	Non-coding	MT-D Loop
709	G	G	G	**A**	G	rRNA	MT-RNR1
750	A	**G**	**G**	**G**	**G**	rRNA	MT-RNR1
1438	A	**G**	**G**	**G**	**G**	rRNA	MT-RNR1
1811	A	A	A	A	**G**	rRNA	MT-RNR2
1888	G	G	G	**A**	G	rRNA	MT-RNR2
2706	A	A	A	**G**	**G**	rRNA	MT-RNR2
3010	G	**A**	G	G	G	rRNA	MT-RNR2
3316	G	G	G	**A**	G	Ala4Thr	MT-ND1
4216	T	T	T	**C**	T	Tyr304His	MT-ND1
4336	T	T	**C**	T	T	tRNA	MT-TQ
4646	T	T	T	T	**C**	Tyr59Tyr	MT-ND2
4769	A	**G**	**G**	**G**	**G**	Met100Met	MT-DN2
4917	A	A	A	**G**	A	Asn150Asp	MT-DN2
5999	T	T	T	T	**C**	Ala32Ala	MT-CO1
6047	A	A	A	A	**G**	Leu48Leu	MT-CO1
7028	C	C	C	**T**	**T**	Ala375Ala	MT-CO1
7258	T	T	T	**C**	T	Ile452Thr	MT-CO1
7705	T	T	T	T	**C**	Tyr40Tyr	MT-CO2
8308	A	A	A	A	**G**	tRNA	MT-TK
8697	G	G	G	**A**	G	Met57Met	MT-ATP6
8860	A	**G**	**G**	**G**	**G**	Thr112Ala	MT-ATP6
9377	A	**G**	A	A	A	Trp57Trp	MT-CO3
9389	A	A	A	A	**G**	Val61Val	MT-CO3
10321	T	T	T	**C**	T	Val88Ala	MT-ND3
10463	T	T	T	**C**	T	tRNA	MT-TR
10819	A	A	A	A	**G**	Lys20Lys	MT-ND4
11251	A	A	A	**G**	A	Leu164Leu	MT-ND4
11332	C	C	C	C	**T**	Ala191Ala	MT-ND4
11467	A	A	A	A	**G**	Leu236Leu	MT-ND4
11719	G	G	G	**A**	**A**	Gly320Gly	MT-ND4
12307	A	A	A	A	**G**	tRNA	MT-TL2
12308	A	A	A	A	**G**	tRNA	MT-TL2
12372	G	G	G	G	**A**	Leu12Leu	MT-ND5
12633	C	C	C	**A**	C	Ser99Ser	MT-ND5
13203	A	**G**	A	A	A	Ala289Ala	MT-ND5
13368	G	G	G	**A**	G	Gly344Gly	MT-ND5
13528	A	A	A	A	**G**	Thr398Ala	MT-ND5
13565	C	C	C	C	**T**	Ser410Phe	MT-ND5
14279	G	**A**	G	G	G	Ser132Leu	MT-ND6
14620	C	C	C	C	**T**	Gly18Gly	MT-ND6
14766	C	C	C	**T**	**T**	Thr7Ile	MT-CYB
14905	G	G	G	**A**	G	Met53Met	MT-CYB
15326	A	**G**	**G**	**G**	**G**	Thr194Ala	MT-CYB
15373	A	A	A	A	**G**	Leu209Leu	MT-CYB
15452	C	C	C	**A**	C	Leu236Ile	MT-CYB
15607	A	A	A	**G**	A	Lys287Lys	MT-CYB
15693	T	T	T	T	**C**	Met316Thr	MT-CYB
15758	A	A	A	A	**G**	Ile338Val	MT-CYB
15833	C	C	**T**	C	C	Leu363Leu	MT-CYB
15928	G	G	G	**A**	G	tRNA	MT-TT
16126	T	T	T	**C**	T	Non-coding	MT-DLOOP
16163	A	A	A	**G**	A	Non-coding	MT-DLOOP
16304	T	T	**C**	T	T	Non-coding	MT-DLOOP
16519	T	**C**	T	**C**	**C**	Non-coding	MT-DLOOP

## Discussion

This clinico-pathological study demonstrates the range of clinical and pathological phenotypes that can be caused by the same homozygous mutation in a nuclear gene controlling the integrity of the mitochondrial genome (Table 3). We provide extensive genetic studies to support the contention that it is likely that the mtDNA ‘*phenotype’* (caused by somatic changes resulting from impaired mtDNA maintenance and proofreading) determines the remarkable variation in clinical and pathological phenotypes, rather than the underlying nuclear *POLG* mutation, which is identical in each of the cases presented. One extreme example of this hypothesis is the early onset and severe disease associated with mtDNA depletion as compared with multiple mtDNA deletions. The reason why mtDNA is depleted in some patients whereas mtDNA deletions and somatic point mutations accumulate in others remains obscure.

**Table 3:** The mitochondrial phenotypes and clinical features reported with homozygous A467T mutations.

PHENOTYPE	CLINICAL FEATURES	REFERENCE
PEO	Progressive external ophthalmoplegia; seizures	[7]
SANDO	Sensory ataxia; dysarthria; ophthalmoparesis	[26]
Alpers Huttenlocher syndrome	Epilepsy; EPC; psychomotor regression; liver failure; neuropathy; range of onset from 1–36 years	[3, 27, 28]
Encephalopathy	Encephalopathy; stroke-like episodes; myoclonus; PEO	[3]
MEMSA	Myopathy, epilepsy, and ataxia without ophthalmoplegia.	[3]
“MNGIE-like”	Gastro-intestinal dysmotility; cachexia; PEO; ptosis; peripheral neuropathy; no leukoencephalopathy; normal plasma thymidine	[29]
Ataxia	Hypotonia, Headache, muscle weakness	[7]
Epilepsy	Ataxia, myoclonic seizures, optic atrophy, dysarthria, and developmentally delayed	[3, 7]
Epilepsy with occipital lobe predilection	SPS; CPS; sGTCS; SE Ataxia; headache; vomiting;	[30]
“Mitochondrial ataxia +” syndrome	Ataxia; migraine-like headaches; focal epilepsy; myoclonus; PEO; neuropathy	[31, 32]

Abbreviations: CPS, complex partial seizure; EPC, epilepsia partialis continua; MEMSA, Myoclonic epilepsy myopathy sensory ataxia; MINGIE, mitochondrial neurogastrointestinal encephalopathy; PEO, progressive external ophthalmoplegia; SE, status epilepticus; sGTCS, secondary generalised tonic-clonic seizures; SPS, simple partial seizure.

Despite harbouring identical genetic mutations, all four individuals exhibited striking clinical heterogeneity and confirm the extensive range of neurological involvement associated with homozygous A467T mutations. Several points in relation to their clinical features merit comment. Patient 1 was the most severely affected, with a clinical diagnosis of Alpers-Huttenlocher syndrome. The neuropathological findings confirm those of Alpers-Huttenlocher syndrome with the additional finding of bilateral hippocampal sclerosis (ILAE Type 1) [17]. Although most cases of Alpers-Huttenlocher syndrome are due to compound heterozygote mutations in *POLG*, our findings confirm that homozygous A467T mutations are also an important cause. Patient 2’s presentation falls within the “MEMSA+” spectrum with an initial encephalopathic episode followed by the subsequent development of cortical blindness, myoclonus, ataxia, myopathy and neuropathy. However, her disease course was atypical in that following her initial presentation she remained stable, seizure-free and essentially asymptomatic (other than the visual sequelae arising from her initial presentation) for a number of years before deteriorating further. In addition, she also had titubation and a tremor, which are not commonly reported with A467T mutations. Patient 3 presented with a classical picture of SANDO whilst Patient 4, who had the mildest phenotype of these four cases, was previously diagnosed as having “MELAS”.

Although we have assigned diagnostic labels such as “Alpers-Huttenlocher”, “MEMSA”, “SANDO” and “MELAS-like” to emphasise the salient features of their phenotypes, it is clear that there is considerable clinical overlap between all four patients. Following a review of all published A467T mutations in the literature, and utilising the Human DNA Polymerase γ Database, we compiled a table of the phenotypic spectrum of homozygous A467T-related mitochondrial disease (Table 3). Thus, our findings are in accord with the prevailing view that the neurological features arising from the A467T mutation lie on a continuum, rather than representing discrete and circumscribed clinical syndromes [18].

Long-range PCR analysis of mtDNA derived from the muscle tissue of all four cases demonstrated depletion of mtDNA in patient 1 consistent with previous reports of Alpers-Huttenlocher syndrome, and multiple deletions of mtDNA in patients 2, 3 and 4. The mechanism by which genetic defects in *POLG* induce such changes in mtDNA is still unclear. It is likely that recessively-inherited mutations cause disease via a loss of function effect, whilst dominantly acting heterozygous mutations produce an inactive form of the enzyme that competes with wild-type pol γ. As a result, mtDNA replication is impaired and error-prone, allowing introduction of point mutations and/or deletions of mtDNA molecules or progressive depletion of mtDNA copy number [19]

Pol γ is a heterotrimer consisting of a single 140 kDa catalytic subunit, Pol-γ A, and a tight dimer of an accessory subunit, Pol-γ B. Pol-γ A consists of three domains: an N-terminal domain containing 3’→5’ exonuclease, a spacer domain, in which the A467T mutation is located and a C-terminal domain, containing 5’→3’ DNA polymerase activity. The accessory subunit enhances DNA binding affinity and processivity. Chan and colleagues reported a profound reduction in polymerase activity by the A467T mutant enzyme with a failure to interact with the accessory subunit resulting in impaired DNA binding and processivity [20] [13]. However, other pathogenic mutations in the spacer region exhibited DNA-binding affinities and processivities similar to normal controls [11] suggesting that other unknown factors beyond the basic catalytic functions of Pol γ are also likely to influence the disease-causing mechanisms. More recently, Euro and colleagues used the recently solved crystal structure of human Pol γ [20] to analyse the structure-function relationships of various recessive mutations associated with Alpers-Huttenlocher syndrome [21]. In addition to demonstrating reduced DNA-binding affinity and Pol γ activity, they predicted that A467T would disrupt the hydrophobic structure of the spacer domain, thus impairing the function of the enzyme. The authors postulated that, as most patients with Alpers-Huttenlocher syndrome were compound heterozygotes for *POLG* mutations, their phenotype could be explained by the functional clusters (designated 1 to 5) to which each mutation belonged. Moreover, A467T belonged to cluster 2 and according to their analysis, would cause Alpers-Huttenlocher syndrome if associated with a mutation from clusters, 1, 3, 4 and 5. Thus, their model does not account for why homozygous A467T mutations would result in the severe Alpers-Huttenlocher phenotype observed in patient 1.

The interplay between mtDNA variants and mutations in nuclear genes may in part explain the variation in mitochondrial phenotypes. To investigate this possibility further we undertook resequencing of mtDNA using the MitoChip tool, which is able to detect levels of heteroplasmy as low as 2% [22]. Interestingly, each of the four patients had a different mtDNA haplotype (Table 2), raising the possibility that the mtDNA haplotype influences the *POLG* mutation-related phenotype, although clearly a much larger cohort would be needed to study this in more detail. One key question is whether the clinical heterogeneity in part arises from existing mtDNA variation or whether misreplication from the A467T mutant enzyme results in the variability in mtDNA which in turn influences the phenotype. Mitochondrial DNA resequence analysis demonstrated a number of sites of polymorphisms in the four patients, which varied considerably between patients. We identified a G to A mutation at np 14279 in the *ND6* gene in patient 1. Although patient 1 did not exhibit any ocular involvement, this mutation has been previously reported in a family with Leber’s hereditary optic neuropathy [23]. However the potential pathogenic role of this mutation remains unclear especially since the mutated residue is not highly conserved across species.

Patient 4 harboured a number of rare variants in her mtDNA, including two missense mutations: an A>G point mutation at np 13528 and a C>T change at np 13565 in the complex 1 *ND5* gene, both of which have been previously reported in this patient [16]. Interestingly both mutations have been described in haplogroup U in the absence of mitochondrial disease [24]. Although both mutations were homoplasmic or near homoplasmic in the patient’s muscle and blood, the m.13528A>G mutation was heteroplasmic in the patient’s asymptomatic mother suggesting the possibility that an increase in the mutant mtDNA load in the patient may have influenced her phenotype. However this is speculation. The contributions of both variants to the disease phenotype remains unclear. Fibroblasts from the patient exhibited decreased mitochondrial membrane potential and increased lactate production, consistent with impaired mitochondrial function. Finally, patient 4 also harboured a G>A mutation at np 12307. This has never previously been reported in either individuals with mitochondrial disease or normal controls according to a current review of mtDNA databases. Thus its functional role remains uncertain. The further finding of the A467T *POLG* mutation in the same individual raises a few important points. First, our findings confirm that the “MELAS” phenotype can be caused by mutations in *POLG*, which is important in the differential diagnosis of this condition. Second, the question arises as to whether patients who have previously been reported to harbour mitochondrial diseases typically associated with mtDNA point mutations should undergo *POLG* sequencing to exclude a nuclear gene defect as the cause of their phenotype [25], particularly if there is a Mendelian pattern of inheritance. Third, from a mechanistic point of view, it is possible that such mtDNA variations caused by defective Pol γ activity as a result of mutations in *POLG* may influence the overall mitochondrial phenotype.

In summary, our data provides evidence that the phenotype caused by a homozygous nuclear gene mutation, A467T in *POLG*, is strongly related to the downstream mtDNA effects in an individual patient, so that mtDNA depletion results in an early-onset severe phenotype, whereas deletions are associated with later onset disease, and in one case fixation of a heteroplasmic mtDNA point mutation (arguably the result of defective Pol γ proofreading activity) may have contributed to a MELAS-like phenotype.
